# Metagenomic Sequence of Prokaryotic Microbiota from an Intermediate-Salinity Pond of a Saltern in Isla Cristina, Spain

**DOI:** 10.1128/genomeA.00045-14

**Published:** 2014-02-13

**Authors:** Ana B. Fernández, María José León, Blanca Vera, Cristina Sánchez-Porro, Antonio Ventosa

**Affiliations:** Department of Microbiology and Parasitology, Faculty of Pharmacy, University of Seville, Seville, Spain

## Abstract

Marine salterns are artificial multipond systems designed for the commercial production of salt by evaporation of seawater. We report here the metagenomic sequence of the prokaryotic microbiota of a pond with intermediate salinity (21% total salts) of a saltern located in Isla Cristina, Huelva, southwest Spain.

## GENOME ANNOUNCEMENT

Marine salterns are composed of interconnected ponds with increasing salt concentrations and they constitute excellent models for studying microbial populations growing along a salinity gradient (1). They are used for the commercial production of salt by evaporation of seawater (2). Microbiological studies of these extreme habitats have enabled identification of the microorganisms inhabiting them (3–5). However, most studies have been focused on the most concentrated hypersaline ponds, designated crystallizers, in which the NaCl is precipitated (6–8). Molecular studies have shown that the two main prokaryotes which are predominant in these NaCl-saturated ponds are the haloarchaeon *Haloquadratum walsbyi* and the bacteroidete *Salinibacter ruber* (9, 10). Recent metagenomic studies carried out in the salterns in Santa Pola, east Spain, have confirmed these findings (11). However, in contrast to the findings of reduced microbial diversity in the highly saline ponds (crystallizers), analysis of the metagenomic data set of an intermediate-salinity pond with 19% salts indicated that a much wider prokaryotic diversity than expected was present (11). In this report, we describe the sequencing of a prokaryotic metagenome obtained from a pond of intermediate salinity of a marine saltern located in Isla Cristina, Huelva, southwest Spain, on the Atlantic Ocean, far away from the Santa Pola saltern, which is on the Mediterranean coast.

A 50-liter water sample was obtained from the surface of a pond with 21% total salts (Global Positioning System [GPS] coordinates 37°13′08″N, 7°20′17″W) on July 2010. The sample was sequentially filtered through 5.0- and 0.22-µm-pore size polycarbonate filters (Millipore) as previously described (11). Total environmental DNA was extracted and purified following the protocols described elsewhere (11, 12). The purified prokaryotic DNA was sequenced by pyrosequencing (Roche 454 GS-FLX system, Titanium chemistry) by GATC, Constance, Germany. A total of 1,223,923 reads with an average read length of 397 bp were obtained. The size of the metagenomic data set was 486,317,437 bp.

Sequences related to 16S ribosomal RNA genes were identified using the Ribosomal Database Project (RDP) (13). In addition, sequences were compared against the MG-RAST server (14), Community Cyberinfrastructure for Advanced Microbial Ecology Research and Analysis (CAMERA) (15), and Integrated Microbial Genomes with Microbiome Samples-Expert Review (IMG/MER) (16) databases.

*Euryarchaeota* (~84%) was the most abundant phylum present in this sample, followed by *Bacteroidetes* (~8%) and *Gammaproteobacteria* (~7%), suggesting a decrease of the microbial diversity in salt ponds of salterns when the salinity reaches salt concentrations >20%, being more similar to those found in crystallizer ponds than those found in intermediate-salinity ponds with <20% salts.

Further metagenome analysis will contribute increased understanding of the genetic diversity present in marine salterns and particularly in ponds with intermediate salinities where the environmental conditions limit the presence of halophilic bacteria and enhance the growth of haloarchaea.

### Nucleotide sequence accession number.

The sequences obtained in this project have been deposited in the NCBI Sequence Read Archive under the accession no. SRP029970.
